# Effect of protein and carbohydrate solutions on running performance and cognitive function in female recreational runners

**DOI:** 10.1371/journal.pone.0185982

**Published:** 2017-10-12

**Authors:** Zhaohuan Gui, Fenghua Sun, Gangyan Si, Yajun Chen

**Affiliations:** 1 Department of Maternal and Child Health, School of Public Health, Sun Yat-Sen University, Guangzhou, Guangdong, China; 2 Department of Health and Physical Education, The Education University of Hong Kong, Tai Po, Hong Kong; University of Birmingham, UNITED KINGDOM

## Abstract

This study compared the effects of a carbohydrate–electrolyte–protein solution (CEPS, 2% protein plus 4% carbohydrate), carbohydrate–electrolyte solution (CES, 6% carbohydrate), and noncaloric sweetened placebo (PLA) on both 21-km running performance and cognitive function. Eleven female recreational endurance runners performed a 21-km time-trial running on three occasions, separated by at least 28 days. In a randomized cross-over design, they ingested CEPS, CES, or PLA at a rate of 150 mL every 2.5 km with no time feedback. A cognitive function test was performed before and after the run. Participants ingested approximately 24 g/h carbohydrate plus 12 g/h protein in CEPS trial, and 36 g/h carbohydrate in CES trial during each 21-km trial. Time to complete the time-trial was slightly shorter (*P* < 0.05) during CES (129.6 ± 8.8 min) than PLA (134.6 ± 11.5 min), with no differences between CEPS and the other two trials. The CEPS trial showed higher composite of visual motor speed than the PLA trial (*P* < 0.05). In conclusion, CES feedings might improve 21-km time-trial performance in female recreational runners compared with a PLA. However, adding protein to the CES provided no additional time-trial performance benefit. CEPS feeding during prolonged exercise could benefit visual motor speed compared to PLA alone, but no differences in the performance of the other cognitive function tests were found.

## Introduction

The preponderance of research on carbohydrate–electrolyte solution (CES) consumption during endurance exercise has shown that exercise performance is improved and fatigue is delayed compared with a noncaloric placebo (PLA) or water [1–3], likely via maintenance of euglycemia and a high rate of carbohydrate (CHO) oxidation [4,5]. Because there appears to be an upper limit to exogenous CHO oxidation mediated by absorption mechanisms [4], it has been hypothesized that the addition of other macronutrients to a CHO drink can further improve performance. Recently, the addition of protein (PRO) to the CES (CHO–electrolyte–PRO solution, CEPS) has been suggested to further improve exercise capacity compared with the CES alone [3,6,7]. These studies utilized cycling time to exhaustion (TTE), which, although a frequent measure of performance, has shown a poor reproducibility [8]. In contrast, the exercise protocols that require a set amount of work to be completed as quickly as possibly (i.e. a time-trial) or involve the accomplishment of the greatest amount of work in a set period of time are closer to the competitive task and are more reproducible. Several other studies have also assessed the impact of CEPS on time-trial performance, yet no studies reported additional improvement in time-trial when PRO added to the CES [8–10]. However, the CEPS trial contained about 20% to 37% more calories than the CES-only trial in these studies [8–10]. While a generic effect of adding calories during prolonged exercise has been widely acknowledged, additional calories can slow down gastric emptying, thus, could put the participants at a greater gastrointestinal distress wherein effort may have been diminished [11]. Furthermore, participants received a high rate of CHO (60g/h) feeding during exercise in their research [8, 10]. It has been suggested that when CHO is ingested at levels that approach the maximal rate of exogenous glucose oxidation of approximately 60–70 g/h [5], the addition of PRO to a CES does not further enhance performance [10]. Thus, it is necessary to investigate the effect of CEPS consumption on exercise performance when total CHO and energy intake during exercise is not so high. This would be particularly beneficial for athletes and individuals who are concerned about caloric intake during training or potential gastrointestinal problems. Theoretically, the addition of PRO to the CES may facilitate faster fuel-medium transport across the lining of the intestine [12] and result in a greater insulin response [3], which may benefit the endurance performance. Additionally, energy matching between trials could be better to evaluate whether the combination of exogenous CHO and PRO would provide more benefits to endurance exercise, compared with CHO ingestion alone. Sex-based differences in substrate metabolism and feeding tolerance during endurance exercise have been well established. Specifically, women generally oxidize lower proportions of CHO than men, but experience more gastrointestinal symptoms during a bout of endurance exercise [13,14]. Sex also influences PRO oxidation during exercise, particularly of the branched chain amino acid leucine. In comparison to men, premenopausal women oxidize less leucine during endurance exercise [15]. Furthermore, non-oxidative leucine disposal is greater in women (reflective of whole-body protein synthesis) during endurance exercise than in men [15]. Taken together, the findings from sex comparative studies show that women rely to a lesser extent on CHO and PRO sources during endurance exercise. In addition, women in the luteal phase have a lesser reliance on CHO sources to fuel endurance exercise compared with women in the follicular phase [13], re-enforcing the importance of standardizing the menstrual status in such kind of studies. Despite the well-recognized sexual dimorphisms in physiologic responses during exercise, there is a paucity of research examining the effects of CEPS aimed to enhance performance in women.

There is also mounting evidence that CHO feedings can ameliorate the cognitive dysfunction that occurs following exercise [16–18]. The term “cognitive function” describes the performance of objective tasks that require conscious mental effort [19]. Such tasks require (verbal, spatial, and working) memory, attention, and executive control [20]. In many sports, participants have to simultaneously perform physically demanding mechanical work and a decisional or perceptual task. Altered cognitive function may affect mood, motivation, the processing of incoming somatosensory information, perceived exertion, and the excitability of the motor cortex, and then impaired voluntary performance during exercise [21–23]. The reduction in cognitive function following prolonged exercise are associated with some nutrition metabolic responses, i.e., low blood glucose and high free fatty acid concentration [24,25]. CHO supplementation could potentially enhance cognitive performance by increasing cerebral glucose uptake and oxygen consumption [26], reducing ammonia production [24], limiting the transport of tryptophan into the brain, and presumably 5-hydroxytryptamine synthesis [25]. However, there are also studies show that CHO ingestion did not improve cognitive function during endurance exercise [27,28]. These discrepancies may arise from the use of different exercise protocols and/or CHO consumption protocols. Regardless, further study is warranted to better clarify the role of CHO in cognitive function.

A consistent beneficial effect of PRO supplementation on cognitive function has been observed across different populations under resting conditions [29,30]. However, to date, the data is still limited examining the effect of PRO or CEPS ingestion during prolonged exercise on cognitive function. This is unfortunate because endurance performance may be affected by decreased cognitive function when athletes are tired.

Therefore, the purpose of this study was to investigate whether the CEPS would improve running performance and cognitive function, as compared with a CES and a PLA in female recreational marathon runners. We hypothesized that CEPS feeding would improve the endurance performance and attenuate the expected reductions in cognitive performance induced by prolonged exercise compared with a CES-only drinks and a PLA.

## Materials and methods

### Participants

Eleven female recreational runners (age: 32.4 ± 6.7 years, body mass index: 21.0 ± 2.1 kg/m^2^, and maximal oxygen uptake (*V*O_2max_): 49.0 ± 6.6 mL/kg/min; mean ± SD) volunteered to participate in this study. Inclusion criteria for the study included a self-reported weekly running frequency > 3 days/week over the preceding 2 months and all participants had at least one marathon race experience. The purpose and potential risks of the experiment were explained to them before participation. They all signed a written consent form and completed a menstrual cycle questionnaire to determine the length of their menstrual cycle. The present study was approved by the Ethics Committee of The Education University of Hong Kong.

### Study design

All participants performed three main experimental trials in a randomized, double-blinded, counterbalanced manner, at intervals of at least 28 days. In each main trial, participants consumed one of three solutions, namely the CES (6% CHO), CEPS (4% CHO + 2% PRO), and PLA, at a rate of 150 mL every 2.5 km during a 21-km time-trial run. The running performance and cognitive function were recorded after the run.

### Preliminary tests

Before the main experimental trials, all participants reported to the laboratory for the assessment of their *V*O_2max_ and for familiarization with the exercise protocol. They completed a series of preliminary tests to determine the following: 1) the relationship between oxygen uptake (*V*O_2_) and submaximal running speed through a 16-min incremental test on a level treadmill and 2) the *V*O_2max_ through an uphill incremental treadmill running test to volitional exhaustion, as described by Williams et al [31]. On the basis of the two preliminary test results, a running speed equivalent to 70% of each participant’s *V*O_2max_ was determined. Thereafter, participants completed a familiarization trial to confirm the running speeds equivalent to 70% of the individual *V*O_2max_. This speed was used in the first 5 km of the 21-km main trials. All participants were also instructed to complete three cognitive function tests to become familiar with the test battery and to minimize the learning effect before the main trials.

### Physical activity, nutrition, and menstrual status control

All participants were instructed to maintain their aerobic exercise as regularly as possible for the duration of the experiment in order to minimize variance in physical condition among trials. For 48 h before each main experiment trial, the participants were asked not to perform strenuous and unaccustomed physical activities. Participants were also instructed to record their food and drink consumption for 48 h before the first main trial and repeat the same diet before each subsequent trial, as well as to refrain from alcohol or caffeine consumption 24 h before main trial. In addition, all participants completed three main experimental trials at intervals of at least 28 days to standardize the menstrual cycle phase; the trials were usually finished within 10 days after menses ended. To offset any potential effects, each experiment was conducted at the same time of the day (e.g., 9:00 am, 12:00 pm, or 2:00 pm). The participants were instructed to consume at least 500 mL of water 2 h before arriving at the laboratory to ensure that they were normally hydrated before the main trials. Constant temperature (22°C) and relatively humidity (60%) were maintained throughout the experiment by a thermostat.

### Experimental trials

Upon arriving at the laboratory, participants assumed a seated position and rested for 30 min. After baseline data collected, a standardized 5-min warm-up was performed at 6 km/h. Then, the speed of the treadmill was immediately increased to the intensity of 70% of the individual *V*O_2max_ until participants completed the first 5 km. Thereafter, the participants ran at whatever speed they wished for the remaining 16 km of the performance run [32]. They could freely alter the speed of the treadmill at any time throughout the trials by using two buttons on the treadmill. To ensure maximal effort during the run, all participants received constant verbal encouragement. The only feedback that participants received during the time-trial was distance covered, which was displaced in the corner of the treadmill screen. Every 2.5 km throughout the run, 150 mL of one of the three solutions was randomly provided to the participants in an opaque cup covered by a lid, and participants commenced drinking from the onset of the time-trial. Both the participant and the investigator were blind to the contents of the solutions. The three solutions were formulated according to Aquarius (Coca-Cola, HK), and they contained the same electrolyte profile and were similarly flavored. The only difference among the three beverages was that the CES contained 6% CHO in the form of sucrose, CEPS contained 4% CHO plus 2% whey PRO (bcshop, HK), and PLA was a noncaloric artificially sweetened solution. The total energy was matched between the CES and CEPS (Table 1). In the CES trial, the CHO ingestion rate was ~36g/h, whereas in CEPS trial, the CHO and PRO ingestion rates were ~24g/h and ~12g/h, separately.

**Table 1:** Composition of experiment beverages.

	Supplements		
Ingredient (per 100 mL)	CEPS	CES	PLA
Carbohydrate (g)	4	6	0
Protein (g)	2	0	0
Sodium (mg)	30	30	30
Potassium (mg)	7	7	7
Magnesium (mg)	1.1	1.1	1.1
Calcium (mg)	0.7	0.7	0.7
Kilocalories	24	24	0

CEPS: carbohydrate-electrolyte-protein solution; CES: carbohydrate-electrolyte solution; PLA: placebo.

### Data collection and sample analysis

**Fig 1:**
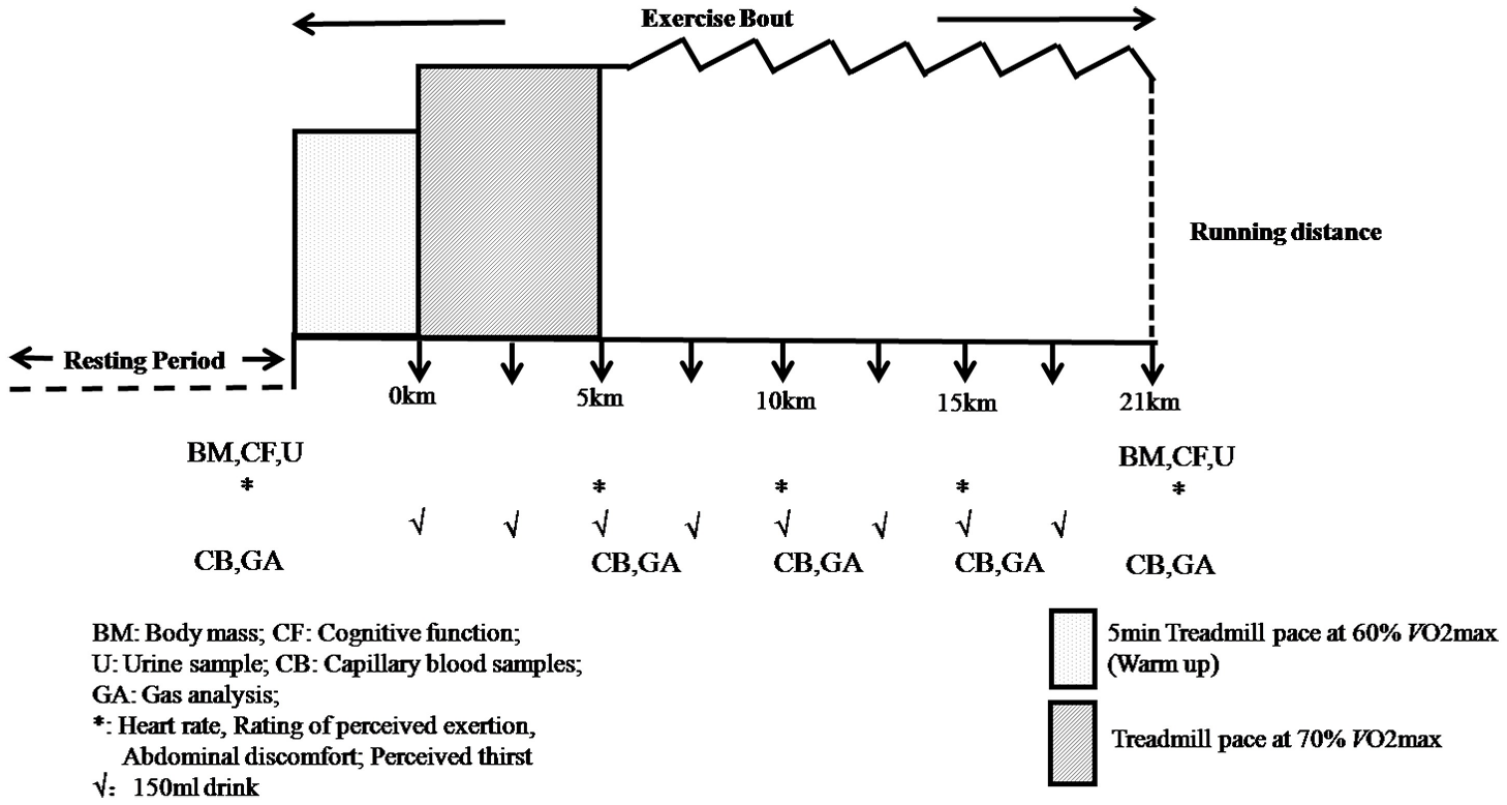
Schematic representation of experimental protocol.

Data collection procedures are illustrated in Fig 1. The body mass (in underwear only) was measured to the nearest 0.1 kg before and after exercise using a weighing machine (Body Weight Precisa, DPS-Promatic, Forli, Italy). The *V*O_2_, carbon dioxide production (*V*CO_2_), and respiratory exchange rate (RER) were measured every 5 km throughout the exercise protocol by using a metabolic cart system (Cortex Metalyzer II-R, CORTEX, Germany). The heart rate (HR) was continuously recorded during the running using a Polar HR monitor (PolarTeam System, Polar Electroy, Finland). Subjective measures, such as a subjective rating of perceived exertion (RPE), perceived thirst (PT), and abdominal discomfort (AD), were recorded just before the gas collection. The RPE was measured using the Borg scale ranging from 6 to 20 [33], and the PT and AD varied from 0 to 10, where 0 denoted “not so much” and 10 denoted “very much” [32]. Capillary blood samples were collected to determine the blood lactate and glucose levels using YSI 1500 (Yellow Spring Instrument Co. Ltd., USA) and a biochemical analyzer (Roche^®^ ACCU-CHEK Reflotron plus, USA), respectively. Urine samples were collected before and after each main experimental trial to measure the urine specific gravity (USG; Atago UG-alpha, Atago Co. Ltd., Tokyo, Japan).

### Cognitive function tests

A battery of cognitive function tests (imPACT Package, imPACT Application, Inc., Australia) [34] was used to provide information on various cognitive parameters before and after exercise. Completion of the entire battery required approximately 20 min. The imPACT battery comprises six tasks—word memory learning, design memory learning, Xs and Os, symbol match, color match, and three letters—which yield the following composite scores: verbal memory, visual memory, visual motor speed, reaction time, and impulse control. The cognitive efficiency index measured the interaction between accuracy (percentage correct) and speed (reaction time) in seconds through the symbol match test. A high score indicated that the participants had done well in both the speed and memory domains of the symbol match test. A low score indicated that the participants had performed poorly in both the speed and accuracy components. In addition, the symptom section included 22 symptoms such as headache, sadness, and fatigue. This symptom score presented the summary information regarding the participant’s self-reported symptom data. A high score reflected a higher symptom total. All of the tasks were fully supervised, and brief onscreen instructions were provided. Responses were recorded on the computer.

### Statistical analysis

Statistical analysis was performed using SPSS software (SPSS 21.0, IBM, USA). Variable that consisted of a single measurement per trial were analyzed using a one-way (trial) repeated-measures analysis of variance (ANOVA). Variables that included multiple measures per trial were examined using a two-way (trial × time) ANOVA. When a significant main effect or interaction was identified, data were subsequently analyzed using a Bonferroni *post hoc* test. The significance level was set at *P* < 0.05. Data are presented as mean ± SD. The omnibus effect size (ES) of the different trials was partial eta square (η^2^) and may be calculated using the following equation: partial η^2^_trial_ = sum of squares trial / sum of Squares trial + sum squares of error. The ES between any two different trials of three trials was *d* and *d* = (mean of experimental group)-(mean of control group) / sum of standard deviation [35]. Cohen defines the partial η^2^ of 0.01, 0.06, 0.14 as small, medium, and large effects, and the threshold of *d* was 0.2, 0.5, 0.8 for small, medium, and large effects, respectively [35].

## Results

### Exercise performance

**Fig 2:**
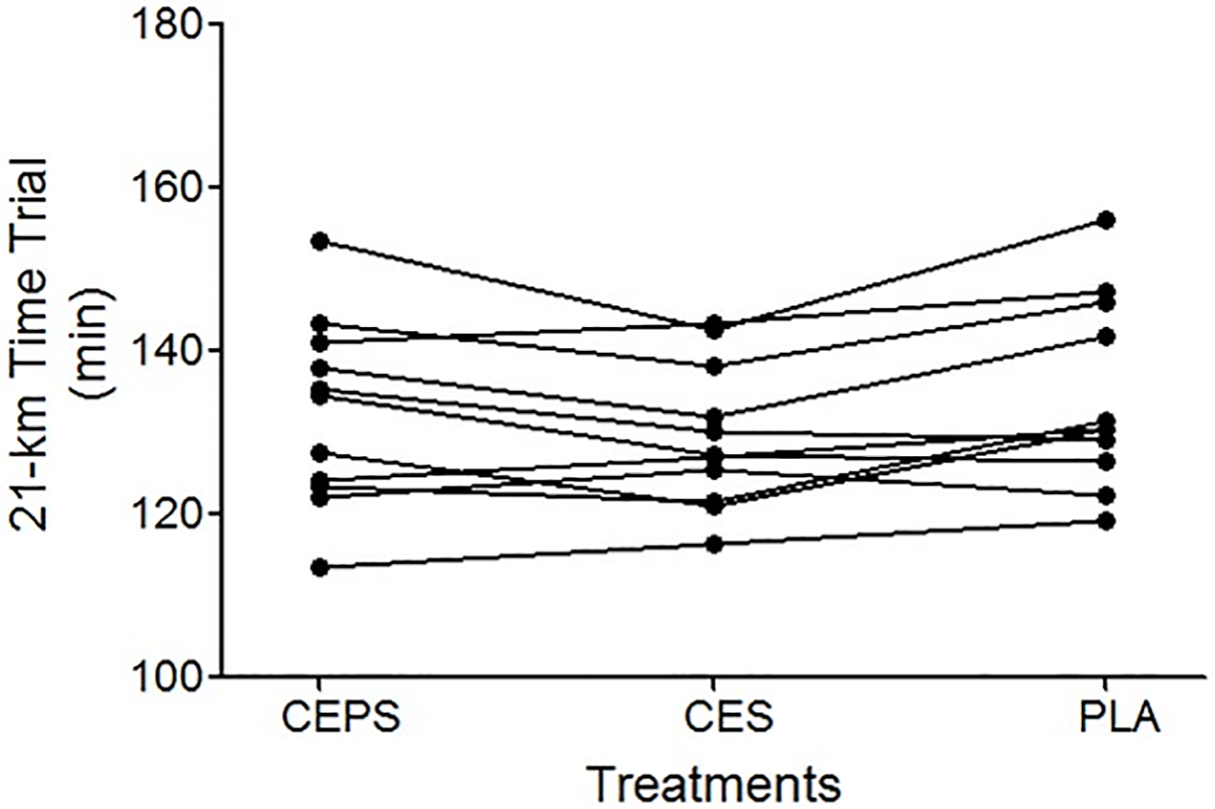
Time required to complete a 21-km marathon run when 11 participants ingested a carbohydrate-electrolyte-protein solution (CEPS), carbohydrate-electrolyte solution (CES), or a placebo (PLA) during exercise.

Average time to complete the 21-km time-trial was 132.4 ± 11.5 min, 129.6 ±8.8 min, and 134.6 ± 11.5 min for the CEPS, CES, and PLA trials, respectively. The endurance performance was approximately 3.7% shorter in the CES trial than in the PLA trial (*P* < 0.05), but no differences were observed between the CEPS and the other two trials (*P* > 0.05). The omnibus ES of the three trials on endurance performance was large (partial η^2^ = 0.34), and the ES for CEPS and CES trials, CEPS and PLA trials, and CES and PLA trials was 0.27, 0.19, and 0.49, respectively. Compared with the CEPS and PLA trials, 7 or 8 of 11 participants performed better in the CES trial. In addition, 9 of 11 participants posted faster times in the CEPS trial than in the PLA trial (Fig 2).

### Levels of hydration and physiological measures

Participants lost approximately 2.0% of their pre-exercise body mass (CEPS vs. CES vs. PLA: 2.20% ± 1.72% vs. 2.23% ± 1.20% vs. 1.75% ± 1.57%) after the 21-km run. Average body mass loss was not significantly different among the three trials (*P* > 0.05). There was no difference in USG changes (post-exercise–pre-exercise) among the three trials (CEPS vs. CES vs. PLA: −0.004 ± 0.008 vs. −0.003± 0.013 vs. −0.001± 0.010; *P*> 0.05).

Summary data of the physiological measures assessed after ingesting either the CEPS, CES or PLA during the 21-km run are presented in Table 2. The blood glucose was significantly higher in the CES trial than in the PLA trial (*P*< 0.05), but no differences were found between CEPS and other two trials (CEPS vs. CES vs. PLA: 4.97 ± 1.22 vs. 5.53 ± 1.05 vs.4.45 ± 1.01 mmol/L; *P* > 0.05). At the 15-km data collection points, the glucose level was higher in the CEPS trial than in the PLA trial (*P*< 0.05). Blood lactate rose significantly in the CEPS and CES trials (*P*< 0.05). No differences in blood lactate were identified among the three trials (CEPS vs. CES vs. PLA: 1.98 ± 0.94 vs. 2.40 ± 1.48 vs. 1.89 ± 0.89mmol/L; *P* > 0.05). HR gradually rose during the course of excise among three trials (*P*< 0.05). No differences in HR were seen among the three trials (CEPS vs. CES vs. PLA: 138 ± 39 vs. 141 ± 40 vs. 134 ± 37 beats/min; *P*> 0.05). There was no difference in RER during exercise among the three trials (CEPS vs. CES vs. PLA: 0.90 ± 0.05 vs. 0.89 ± 0.10 vs.0.86 ± 0.05; *P*> 0.05).

**Table 2:** Summary data for physiological measures during 21-km running while ingesting either CEPS, CES or PLA.

		Main effects of treatments	Pre-exercise	5-km	10-km	15-km	Post-exercise
Glucose (mmol/L)	CEPS	CES vs. PLA (*P*<0.05)	5.81 ± 1.65	4.83 ± 0.87	4.83 ± 1.18	5.01 ± 0.64^a^	4.38 ± 1.23
	CES		5.92 ± 0.95	5.17 ± 0.78^b^	5.49 ± 1.33	5.43 ± 1.06^b^	5.62 ± 1.10^b^
	PLA		5.64 ±0.93	4.33 ± 0.46	4.24 ± 0.87	4.14 ± 0.67	3.88 ± 1.08
Lactate (mmol/L)	CEPS		1.26 ± 0.48	2.04 ± 1.23	2.23 ± 0.96	2.03 ± 0.82	2.34 ± 0.81
	CES		1.26 ± 0.45	1.89 ± 0.78	2.95 ± 1.84	2.61 ± 1.38	3.29 ± 1.64
	PLA		1.28 ± 0.75	1.91 ± 0.76	2.07 ± 0.89	1.69 ± 0.64	1.98 ± 1.20
HR (beats/min)	CEPS		67 ± 13	151 ± 21	153 ± 14	157 ± 17	163 ± 15
	CES		69 ± 13	150 ± 12	163 ± 14	160 ± 16	165 ± 17
	PLA		65 ± 11	146 ± 14	153 ± 14	152 ± 15	155 ± 13
RER (*V*CO_2_/*V*O_2_)	CEPS		0.93 ± 0.09	0.90± 0.05	0.90± 0.05	0.90± 0.05^a^	0.89± 0.05^a^
	CES		0.88± 0.05	0.88± 0.06	0.88± 0.07	0.87± 0.07	0.86± 0.06
	PLA		0.87± 0.05	0.89± 0.04	0.88± 0.03	0.86± 0.04	0.83± 0.05

^a^ *P*<0.05, CEPS vs. PLA;

^b^*P*<0.05, CES vs. PLA.

CEPS: Carbohydrate-electrolyte-protein solution; CES: Carbohydrate-electrolyte solution; PLA: Placebo.

### Subjective measures

Table 3 shows the data for subjective estimates. There were no differences for RPE among the three trials (CEPS vs. CES vs. PLA: 10.1 ± 3.0 vs. 9.9 ± 3.1 vs. 10.4 ± 3.0; *P* > 0.05), PT (CEPS vs. CES vs. PLA: 1.7 ± 1.7 vs. 1.4 ± 1.5 vs. 1.7 ± 1.8; *P* > 0.05), and AD (CEPS vs. CES vs. PLA: 1.0 ± 1.4 vs. 0.8 ± 1.2 vs. 0.9 ± 1.4) (all *P* > 0.05).

**Table 3:** Summary data for psychological parameters during 21-km running while ingesting either CEPS, CES or PLA.

		Main effects of treatments	Pre-exercise	5-km	10-km	15-km	Post-exercise
RPE	CEPS		6.8 ± 0.9	8.8 ± 1.8	10.1 ± 2.2	11.5 ± 2.5	13.3 ± 2.8
	CES		7.0 ± 2.1	9.0 ± 2.5	10.2 ± 2.3	11.2 ± 2.4	12.2 ± 3.5
	PLA		6.9 ± 1.6	9.2 ± 2.1	10.4 ± 1.7	12.0 ± 2.4	13.4 ± 2.3
PT	CEPS		0.4 ± 0.7	1.3 ± 1.3	1.7 ± 1.3	2.4 ± 2.0	2.6 ± 2.3
	CES		0.2 ± 0.6	1.2 ± 1.2	1.6 ± 1.5	2.1 ± 1.8	1.8 ± 1.8
	PLA		0.3 ± 0.7	1.1 ± 1.3	1.5 ± 1.5	2.4 ± 1.8	3.1 ± 2.4
AD	CEPS		0.1 ± 0.3	0.6 ± 1.0	1.4 ± 1.5	1.5 ± 1.4	1.6 ± 1.9
	CES		0.0 ± 0.0	0.3 ± 0.5	0.6± 0.8	1.5± 1.5	1.5± 1.6
	PLA		0.0 ± 0.0	0.7± 1.1	0.7± 1.1	1.2± 1.3	1.8± 1.9

RPE: Rating of exertion; PT: Perceived thirst; AD: Abdominal discomfort;

CEPS: Carbohydrate-electrolyte-protein solution; CES: Carbohydrate-electrolyte solution; PLA: Placebo.

### Cognitive performance

Results of the cognitive function tests are presented in Table 4. For the verbal memory composite (*accuracy*), results showed no difference, irrespective of the fluid intake condition (*P* > 0.05). The omnibus ES of the three trials on verbal memory was medium (partial η^2^ = 0.13), and the ES for CEPS and CES trials, CEPS and PLA trials, and CES and PLA trials was 0.51, 0.84, and 0.35, respectively. The visual memory composite (*accuracy*) was similar among the three trials (*P* > 0.05).The omnibus ES of the trials on visual memory was large (partial η^2^ = 0.14), and the ES for CEPS and CES trials, CEPS and PLA trials, and CES and PLA trials was 0.31, 0.06, and 0.37, respectively. No significant differences in visual motor speed composite (*accuracy*) were found among three trials during the pre-exercise test, but there was a higher visual motor speed in the CEPS trial than in the PLA trial (*P* <0.05). The omnibus ES of the trials on visual motor speed was large (partial η^2^ = 0.37), and the ES for CEPS and CES trials, CEPS and PLA trials, and CES and PLA trials was 0.11, 0.63, and 0.50, respectively. The reaction time was not influenced by the ingested solutions (*P* > 0.05). The omnibus ES of the trials on reaction time was large (partialη^2^ = 0.15), and the ES for CEPS and CES trials, CEPS and PLA trials, and CES and PLA trials was0.09, 0.56, and 0.52, respectively. For impulse control (*errors*), no differences were observed among all three conditions (*P* > 0.05). The omnibus ES of the trials on impulse control was medium (partial η^2^ = 0.06), and the ES for CEPS and CES trials, CEPS and PLA trials, and CES and PLA trials was 0.28, 0.44, and 0.11, respectively. Regarding the total symptom score, the solutions did not show any significant effect (*P* > 0.05). The omnibus ES of the trials on total symptom score was medium (partial η^2^ = 0.04), and the ES for CEPS and CES trials, CEPS and PLA trials, and CES and PLA trials was 0.55, 0.32, and 0.17, respectively. Similarly, cognitive efficiency index showed no significant variation during exercise among the three trials (*P* > 0.05). The omnibus ES of the trials on cognitive efficiency index was large (partial η^2^ = 0.29), and the ES for CEPS and CES trials, CEPS and PLA trials, and CES and PLA trials was 0.46, 0.94, and 0.51, respectively.

**Table 4:** Cognitive performance for different trials (CEPS, CES, and PLA) before and after exercise.

		Main effects of treatments	Pre-exercise	Post-exercise
Verbal memory	CEPS		89.00 ± 14.83	95.64 ± 4.13
	CES		90.18± 12.42	91.09 ± 11.71
	PLA		90.18 ± 8.59	86.46 ± 14.81
Visual memory	CEPS		78.46 ± 12.82	73.55 ± 16.98
	CES		72.00 ± 14.52	78.55 ± 15.53
	PLA		69.36±16.85	72.46 ± 17.02
Visual motor speed	CEPS	CEPS vs. PLA (*P*<0.05)	40.44 ± 4.81	40.18 ± 4.37
	CES		40.71 ± 4.83	39.70 ± 4.43
	PLA		36.90 ± 3.89	37.63 ± 3.76
Reaction time	CEPS		0.68 ± 0.08	0.65 ± 0.08
	CES		0.67 ± 0.11	0.64 ± 0.14
	PLA		0.71 ± 0.12	0.71 ± 0.13
Impulse control	CEPS		3.09 ± 2.30	3.27 ± 2.20
	CES		4.27 ± 4.80	4.27 ± 4.56
	PLA		4.72 ± 4.10	4.18 ± 3.14
Total symptom score	CEPS		2.46 ± 3.72	1.82 ± 2.71
	CES		2.09 ± 2.77	3.36 ± 2.91
	PLA		3.09 ± 4.23	2.82 ± 3.43
Cognitive efficiency index	CEPS		0.32 ± 0.14	0.39 ± 0.18
	CES		0.31 ± 0.14	0.31 ± 0.17
	PLA		0.29 ± 0.13	0.22 ± 0.18

CEPS: Carbohydrate-electrolyte-protein solution; CES: Carbohydrate-electrolyte solution; PLA: Placebo.

## Discussion

This study compared the effects of three different solutions, which were (namely the CES, CEPS, and PLA) consumed during a 21-km run, on the exercise performance and cognitive function in female recreational runners. We observed that compared with the PLA feedings, ingesting CES improved 21-km time-trial performance. However, the addition of PRO to a CES did not further enhance endurance performance. CEPS feeding enhanced visual motor speed compared to the PLA, but no differences in the performance of the other cognitive function tests were found. It is widely accepted that CES ingestion improves endurance performance [1,2]. This was also the case in our experiment, with the CES improving time-trial performance by 3.7% over the non-caloric PLA. As summarized by Coyle and Jeukendrup [4,5], this effect was most likely due to maintenance of blood glucose or a higher rate of CHO oxidation during exercise. In the present study, the blood glucose concentration of PLA treatment was lower than in the CES treatment during exercise. Even though the CEPS treatment contained more calories from PRO and CHO, performance during this treatment improved 1.7% but not statistically different from the PLA. It should to be noted that participants were recreational runners with infrequent exposure to high-intensity exercise in the present study, so a greater variability of fitness level provides a partial explanation for the lack of a significant benefit [36]. In addition, 9 out of 11 participants ran faster in the CEPS trial than in the PLA trial (Fig 2), suggesting that CEPS ingestion might assist endurance performance.

Three previous studies have suggested that ingestion of a CEPS during prolonged exercise extends TTE, compared with a CES only [3,6,7]. However, the practical applications for these works are constrained by the fact that endurance athletes do not typically compete in events that require sustaining a fixed power output for as long as possible. The time-trial protocol in which athletes are required to complete a certain amount of work in the shortest time possible has shown to be more reliable and reproducible for research [8]. Additionally, in these studies, the CEPS contained approximately 20%–25% more calories than the CES supplement [3,6,7]. Therefore, the beneficial effect of the CEPS versus the CES-only supplements could be attributed to the high caloric content of the CEPS rather than a PRO-specific physiological mechanism per se.

Regarding the time-trial performance, our results were consistent with those studies who reported no improvement in performance when PRO was added into the CES [8–10]. The experimental solutions in these 3 studies were matched for total CHO but not for total caloric content. Approximately 20% to 37% more calories was contained in CEPS treatment than their CES-only. Despite this, the cyclists and runners did not perform better with the addition of PRO. Therefore, these studies suggest that the co-ingestion of CHO and PRO during endurance exercise seems not to further improve time-trial performance. Jeukendrup suggested that, to be ergogenic for performance, the optimal intake of CHO was approximately 60-70g/h [5], and a convenient way for athletes to satisfy their hydration needs is to drink 600-1400ml/h solution [10]. However, in our present study, participants received only 600 mL/h fluid drink during the exercise bout and the CHO was provided a rate of around 24g/h in the CEPS trial and around 36g/h in the CES trial. The lower rate of fluid intake and CHO intake could explain at part for the failure of CEPS to improve time-trial performance.

It should be noted that most of studies having examined the effects of CEPS on performance included male participants only [3, 6, 10]. Very few data exist on how women respond to such beverages. Compared with men, women rely to a lesser extent on CHO and PRO sources to fuel a bout of endurance exercise [13,15], which suggested that female may respond differently in a response to a nutritional regimen. In the present study, one limitation was that we did not calculate the CHO and fat oxidation because the indirect calorimetry method was not appropriate for calculation of whole body substrate utilization when PRO was ingested. It has been suggested that trained participants may be more suitable for physical performance test, compared with untrained participants [36]. Therefore, another limitation of the present study was that only recreational female runners were recruited. In summary, the results of the present study indicated that CEPS ingestion during endurance exercise may not further improve time-trial performance in females, compared with iso-caloric CES consumption. In the future, research needs to be conducted using trained females as subjects, and more sophisticated measures of substrate utilization were applied to determine the mechanism.

This study was also designed to determine the impact of CES or CEPS feedings during endurance running on the performance of cognitive function. This had generally not been studied under these conditions but could clearly affect endurance performance. The mechanisms by CES feeding which affect cognitive function have yet to be elucidated, but it is thought to involve the alterations in the cerebral availability of glucose [26], the balance of neurotransmitters such as serotonin and dopamine [25], and/or ammonia production [24]. Previous research examining the role of CES feedings in maintaining cognition during exercise has led to mixed results. Two studies [16,18] and our laboratory [17] have evaluated the effects of CES during prolonged running or team sport exercise and found an improved cognitive performance in CES trial compared with a PLA. Other studies, however, have indicated that there is no benefit of CES to cognitive performance during running and walking exercise compared with the PLA [27, 28], and our results were consistent with these research. Although the PLA trial showed lower blood glucose concentration, the glucose level for the PLA was relatively high, with small differences between the PLA and CES treatments (PLA vs. CES: 4.45 ± 1.01vs. 5.53 ± 1.05mmol/L). That could be an explanation for the failure observation of differences in cognitive function between CES and PLA trials. Several studies have also demonstrated a positive effect of PRO feedings on cognitive function across different populations in controlled settings, but without exercise [29,30]. To our knowledge, the present study may be the first study exploring the cognitive performance of co-ingestion of PRO and CES during endurance exercise. This is obviously important because common occurrences of cognitive dysfunction of athletes during exercise may determine the outcome of a competition. Our data suggest that small amount of PRO to the CES during endurance exercise enhance visual motor speed than the PLA. We speculated that branched-chain amino acids (BCAA) from the PRO might attenuate central fatigue during this trial, because Blomstrand et al. [37, 38] reported improved mental performance with BCAA feeding.

In summary, our results suggest that CES feeding in the form of a 6% CHO during running exercise can improve 21-km time-trial performance in female recreational runners than a non-caloric PLA solution. Nevertheless, adding 2% PRO to a 4% CHO drink showed no additional benefit on endurance performance. The CEPS treatment provided further benefit for visual motor speed than the PLA ingestion only.

## Supporting information

S1 Data The participants’ data for exercise performance and physical measures. (XLSX)

## References

[1] Jeukendrup A, Brouns F, Wagenmakers AJ, Saris WH. Carbohydrate-electrolyte feedings improve 1 h time trial cycling performance. Int J Sports Med. 1997; 18(2): 125–129. doi: 10.1055/s-2007-972607 9081269 10.1055/s-2007-972607

[2] Angus DJ, Hargreaves M, Dancey J, Febbraio MA. Effect of carbohydrate or carbohydrate plus medium-chain triglyceride ingestion on cycling time trial performance. J Appl Physiol. 2000; 88(1): 113–119. 10642370 10.1152/jappl.2000.88.1.113

[3] Ivy JL, Res PT, Sprague RC, Widzer MO. Effect of a carbohydrate-protein supplement on endurance performance during exercise of varying intensity. Int J Sport Nutr Exerc Metab. 2003; 13(3): 382–395. 14669937 10.1123/ijsnem.13.3.382

[4] Coyle EF. Fluid and fuel intake during exercise. J Sports Sci. 2004; 22(1): 39–55. doi: 10.1080/0264041031000140545 14971432 10.1080/0264041031000140545

[5] Jeukendrup AE. Carbohydrate intake during exercise and performance. Nutrition. 2004;20(7–8): 669–677. doi: 10.1016/j.nut.2004.04.017 15212750 10.1016/j.nut.2004.04.017

[6] Saunders MJ, Kane MD, Todd MK. Effects of a carbohydrate-protein beverage on cycling endurance and muscle damage. Med Sci Sports Exerc. 2004; 36(7): 1233–1238. 15235331 10.1249/01.mss.0000132377.66177.9f

[7] Saunders MJ, Luden ND, Herrick JE. Consumption of an oral carbohydrate-protein gel improves cycling endurance and prevents postexercise muscle damage. J Strength Cond Res. 2007; 21(3): 678–684. doi: 10.1519/R-20506.1 17685703 10.1519/R-20506.1

[8] Osterberg KL, Zachwieja JJ, Smith JW. Carbohydrate and carbohydrate + protein for cycling time-trial performance. J Sports Sci. 2008; 26(3): 227–233. doi: 10.1080/02640410701459730 18074296 10.1080/02640410701459730

[9] Coletta A, Thompson DL, Raynor HA. The influence of commercially-available carbohydrate and carbohydrate-protein supplements on endurance running performance in recreational athletes during a field trial. J Int Soc Sports Nutr. 2013; 10(1): 17 doi: 10.1186/1550-2783-10-17 23537142 10.1186/1550-2783-10-17 PMC3614480

[10] Van Essen M, Gibala MJ. Failure of protein to improve time trial performance when added to a sports drink. Med Sci Sports Exerc. 2006; 38: 1476–1483. doi: 10.1249/01.mss.0000228958.82968.0a 16888462 10.1249/01.mss.0000228958.82968.0a

[11] Murray R, Bartoli W, Stofan J, Horn M, Eddy D. A comparison of the gastric emptying characteristics of selected sports drinks. Int J Sport Nutr. 1999; 9(3): 263–274. 10477362 10.1123/ijsn.9.3.263

[12] Shi X, Summers RW, Schedl HP, Flanagan SW, Chang R, Gisolfi CV. Effects of carbohydrate type and concentration and solution osmolality on water absorption. Med Sci Sports Exerc.1995; 27(12): 1607–1615. 8614315

[13] Devries MC, Hamadeh MJ, Phillips SM, Tarnopolsky MA. Menstrual cycle phase and sex influence muscle glycogen utilization and glucose turnover during moderate-intensity endurance exercise. Am J Physiol Regul Integr Comp Physiol. 2006; 291(4): R1120–1128. doi: 10.1152/ajpregu.00700.2005 16690766 10.1152/ajpregu.00700.2005

[14] Wilson PB. Does Carbohydrate Intake During Endurance Running Improve Performance? A Critical Review. J Strength Cond Res.2016; 30(12): 3539–3559. doi: 10.1519/JSC.0000000000001430 27045602 10.1519/JSC.0000000000001430

[15] Lamont LS, McCullough AJ, Kalhan SC. Gender differences in leucine, but not lysine, kinetics. J Appl Physiol. 2001; 91(1): 357–362. 11408452 10.1152/jappl.2001.91.1.357

[16] Collardeau M, Brisswalter J, Vercruyssen F, Audiffren M, Goubault C. Single and choice reaction time during prolonged exercise in trained subjects: influence of carbohydrate availability. Eur J Appl Physiol. 2001; 86(2): 150–156. doi: 10.1007/s004210100513 11822474 10.1007/s004210100513

[17] Wong SH, Sun FH, Huang WY, Chen YJ. Effects of beverages with variable nutrients on rehydration and cognitive function. Int J Sports Med. 2014; 35(14): 1208–1215. doi: 10.1055/s-0034-1370968 25203651 10.1055/s-0034-1370968

[18] Winnick JJ, Davis JM, Welsh RS, Carmichael MD, Murphy EA, Blackmon JA. Carbohydrate feedings during team sport exercise preserve physical and CNS function. Med Sci Sports Exerc. 2005; 37(2): 306–315. 15692328 10.1249/01.mss.0000152803.35130.a4

[19] Lamport DJ, Saunders C, Butler LT, Spencer JP. Fruits, vegetables, 100% juices, and cognitive function. Nutr Rev. 2014; 72(12): 774–789. doi: 10.1111/nure.12149 25399992 10.1111/nure.12149

[20] Lezak MD. Neuropsychological Assessment. NewYork: Oxford University Press; 2004.

[21] Taylor JL, Butler JE, Allen GM, Gandevia SC. Changes in motor cortical excitability during human muscle fatigue. J Physiol. 1996; 490 (Pt 2):519–528. 8821148 10.1113/jphysiol.1996.sp021163 PMC1158688

[22] Davis JM, Bailey SP. Possible mechanisms of central nervous system fatigue during exercise. Med Sci Sports Exerc. 1997; 29(1): 45–57. 9000155 10.1097/00005768-199701000-00008

[23] Nybo L. CNS fatigue and prolonged exercise: effect of glucose supplementation. Med Sci Sports Exerc. 2003; 35(4): 589–594. doi: 10.1249/01.MSS.0000058433.85789.66 12673141 10.1249/01.MSS.0000058433.85789.66

[24] Wagenmakers AJ, Beckers EJ, Brouns F, Kuipers H, Soeters PB, van der Vusse GJ, et al Carbohydrate supplementation, glycogen depletion, and amino acid metabolism during exercise. Am J Physiol. 1991; 260(6 Pt 1): E883–890. 2058665 10.1152/ajpendo.1991.260.6.E883

[25] Davis JM, Bailey SP, Woods JA, Galiano FJ, Hamilton MT, Bartoli WP. Effects of carbohydrate feedings on plasma free tryptophan and branched-chain amino acids during prolonged cycling. Eur J Appl Physiol Occup Physiol. 1992; 65(6): 513–519. 1483439 10.1007/BF00602357

[26] Nybo L, Moller K, Pedersen BK, Nielsen B, Secher NH. Association between fatigue and failure to preserve cerebral energy turnover during prolonged exercise. Acta Physiol Scand. 2003; 179(1): 67–74. doi: 10.1046/j.1365-201X.2003.01175.x 12940940 10.1046/j.1365-201X.2003.01175.x

[27] Ivy JL, Miller W, Dover V, Goodyear LG, Sherman WM, Farrell S, et al Endurance improved by ingestion of a glucose polymer supplement. Med Sci Sports Exerc. 1983; 15(6): 466–471. 6361440

[28] Lee JK, Ang WH, Ng JW, Fan PW, Teo YS, Nolte HW, et al Effects of a carbohydrate-electrolyte solution on cognitive performance following exercise-induced hyperthermia in humans. J Int Soc Sports Nutr. 2014; 11(1): 51 doi: 10.1186/s12970-014-0051-x 25379031 10.1186/s12970-014-0051-x PMC4221684

[29] Kaplan RJ, Greenwood CE, Winocur G, Wolever TM. Dietary protein, carbohydrate, and fat enhance memory performance in the healthy elderly. Am J Clin Nutr. 2001; 74(5): 687–693. 11684539 10.1093/ajcn/74.5.687

[30] Jones EK, Sunram-Lea SI, Wesnes KA. Acute ingestion of different macronutrients differentially enhances aspects of memory and attention in healthy young adults. Biol Psychol. 2012; 89(2): 477–486. doi: 10.1016/j.biopsycho.2011.12.017 22223097 10.1016/j.biopsycho.2011.12.017

[31] Williams C, Nute MG, Broadbank L. Influence of fluid intake on endurance running performance. A comparison between water, glucose and fructose solutions. Eur J Physiol. 1990; 60(2): 112–119. 2335168 10.1007/BF00846030

[32] Wong SH, Siu M, Lok A, Chen YJ, Morris J, Lam CW. Effect of the glycaemic index of pre-exercise carbohydrate meals on running performance. Eur J Sport Sci. 2008; 8(1): 23–33.

[33] Borg GA. Perceived exertion: a note on "history" and methods. Med Sci Sports. 1973; 5(2): 90–93. 4721012

[34] Nelson LD, Pfaller AY, Rein LE, McCrea MA. Rates and Predictors of Invalid Baseline Test Performance in High School and Collegiate Athletes for 3 Computerized Neurocognitive Tests: ANAM, Axon Sports, and ImPACT. Am J Sports Med. 2015; 43(8): 2018–2026. doi: 10.1177/0363546515587714 26059178 10.1177/0363546515587714 PMC4747101

[35] Cohen J. Statistical power analysis for the behavioural sciences (2nd ed). New York: Academic Press; 1988.

[36] Hopkins WG, Schabort EJ, Hawley JA. Reliability of power in physical performance tests. Sports Med. 2001; 31(3): 211–234. 11286357 10.2165/00007256-200131030-00005

[37] Blomstrand E, Hassmen P, Ekblom B, Newsholme EA. Administration of branched-chain amino acids during sustained exercise—effects on performance and on plasma concentration of some amino acids. Eur J Appl Physiol Occup Physiol. 1991; 63(2): 83–88. 1748109 10.1007/BF00235174

[38] Blomstrand E, Hassmen P, Newsholme EA. Effect of branched-chain amino acid supplementation on mental performance. Acta Physiol Scand. 1991; 143(2): 225–226. doi: 10.1111/j.1748-1716.1991.tb09225.x 1962526 10.1111/j.1748-1716.1991.tb09225.x

